# Anterior Chamber Depth and Refractive Change in Late Postoperative Capsular Bag Distension Syndrome: A Retrospective Analysis

**DOI:** 10.1371/journal.pone.0125895

**Published:** 2015-04-24

**Authors:** Min Kyu Yang, Won Ryang Wee, Ji-Won Kwon, Young Keun Han

**Affiliations:** 1 Department of Ophthalmology, Seoul Metropolitan Government-Seoul National University Boramae Medical Center, Seoul, Korea; 2 Department of Ophthalmology, Seoul National University Hospital, Seoul, Korea; 3 Department of Ophthalmology, Myongji Hospital, Kwandong University College of Medicine, Goyang-Si, Gyeonggi, Korea; Massachusetts Eye & Ear Infirmary, Harvard Medical School, UNITED STATES

## Abstract

**Purpose:**

To assess the characteristic findings and effects of laser capsulotomy in patients with late postoperative capsular bag distension syndrome (CBDS).

**Methods:**

Twenty patients diagnosed with late postoperative CBDS between July 2010 and August 2013 were retrospectively reviewed. Before and 1 week after capsulotomy, changes in the anterior chamber depth (ACD) were assessed using ultrasound biomicroscopy. Changes in the refractive status and uncorrected visual acuity (UCVA) were also measured 1 week and 1 month after capsulotomy. For patients who received bilateral cataract surgery, preoperative ACD and axial length measured by IOLMaster were compared between the two eyes.

**Results:**

Twenty-two eyes from 20 patients who had undergone laser capsulotomy showed a mean UCVA improvement of 0.27 ± 0.24 logMAR (range, 0.00–0.90). ACD was increased by an average of +0.04 mm (95% confidence interval, +0.01 to +0.06 mm, p = 0.034), equivalent to predicted refractive change of +0.10 D. The discrepancy between actual (+1.33 D) and predicted refractive change after capsulotomy suggests that refractive change may not be generated from IOL displacement in late postoperative CBDS. Preoperative ACD was deeper in the eye with late postoperative CBDS in all bilaterally pseudophakic patients (mean, 3.68 mm vs. 3.44 mm in the fellow eye, p = 0.068).

**Conclusions:**

Late postoperative CBDS showed refractive changes that were resolved successfully after laser capsulotomy. The convex lens effects of opalescent material in the distended capsular bag may play a major role in myopic shift. A larger preoperative ACD is possibly associated with the development of late postoperative CBDS.

## Introduction

Capsular bag distension syndrome (CBDS) is a rare complication of phacoemulsification with continuous curvilinear capsulorhexis (CCC) and posterior chamber in-the-bag intraocular lens (PC-IOL) implantation. This condition is characterized by the accumulation of an opaque fluid within a closed chamber inside the capsular bag formed by the occluded anterior capsular opening created by the posterior chamber intraocular lens optic [1]. Miyake et al. using the term “capsular block syndrome” instead of CBDS in their article, classified CBDS as intraoperative (CBDS seen at the time of lens luxation following hydrodissection), early postoperative (classical CBDS), and late postoperative (CBDS with liquefied after-cataract or lacteocrumenasia) based on the time of onset [2,3].

Early postoperative CBDS is often accompanied by anterior displacement of the intraocular lens (IOL) optic, resulting in a shallow anterior chamber and myopic shift. Timely treatment of CBDS can correct unwanted myopia, improve visual acuity, and restore the normal anatomic relationships in the eye [4,5]. Thus far, compared to other types of CBDS, late postoperative CBDS is considered to be less frequently associated with a shallow anterior chamber, forward IOL displacement, or myopic change [6,7]. Late postoperative CBDS is often not recognized until the development of posterior capsular opacification (PCO) in many cases and consequently may be underestimated [8].

Late postoperative CBDS can be successfully treated using either Neodymium:Yttrium-Aluminum-Garnet (Nd:YAG) laser capsulotomy or surgical management [6,8,9]. In this report, we present the clinical characteristics and biometric properties of the eyes of patients with late postoperative CBDS who had undergone anterior or posterior Nd:YAG laser capsulotomy.

## Materials and Methods

We retrospectively reviewed cases of late postoperative CBDS in patients treated at Seoul Metropolitan Government-Seoul National University Boramae medical center from July 2010 to August 2013. Late postoperative CBDS was diagnosed by slit lamp examination as a distended posterior capsule with the presence of opaque fluid trapped between the posterior surface of the PC-IOL and the posterior capsule more than 6 months after cataract surgery. Clinical data including preoperative cataract grade, the ocular viscoelastic device (OVD) used, intraocular lens (IOL) type, and refractive status at 2 months after cataract surgery were recorded. Preoperative axial length (AXL) and anterior chamber depth (ACD) were measured using an IOLMaster ultrasound biomicroscope (Carl Zeiss Meditec AG, Jena, Germany) 1 month prior to cataract surgery. The same surgical techniques of uneventful CCC, phacoemulsification, and PC-IOL in-the-bag implantation were used for all patients included in this study. Complete cortex removal and posterior capsule polishing were successfully performed on every patient. Ethical approval was obtained from the Seoul National University_Boramae Hospital International Review Board and patient records/information was anonymized and de-identified prior to analysis. All measurements were taken after informed consent obtained from the patients.

At diagnosis all late postoperative CBDS patients had an ophthalmic examination which included evaluation of uncorrected visual acuity (UCVA) at a distance using a logarithm of the minimum angle of resolution (logMAR) scale, fundus evaluation using an indirect ophthalmoscope, refractive status using an autorefractometer (KR-8100; Topcon corporation, Tokyo, Japan), and intraocular pressure (IOP) using a pneumatic tonometer (CT-80; Topcon corporation, Tokyo, Japan). These evaluations were performed by a single experienced examiner (MK Yang).

AXL and ACD were measured in both eyes before and 1 week after capsulotomy. The AXL was measured using an IOLMaster and the ACD was obtained using a 35-MHz ultrasound biomicroscopy ([UBM], HiScan, Optikon Co. Ltd., Rome, Italy). Using from 5 to 10 consecutive anterior chamber images, ACD was measured as the distance from the inner surface of the cornea to the IOL anterior surface at the corneal center. The measurement was repeated on another day by the same technician and the two results were averaged. When severe decenteration or IOL tilting was observed, the UBM images obtained were not included in the analysis.

When PCO was not present, anterior capsulotomy was considered as a primary treatment option in order to minimize vitreous floaters. If targeting the posterior capsule was difficult due to milky fluid in the capsular bag or a deep-seated posterior capsule in highly myopic eyes, anterior capsulotomy was performed regardless of PCO presence. A small anterior capsular hole was created at 6 o/c using an Nd:YAG laser (Aura PT, Lumenis Ltd., Yokneam, Israel); this was followed by Nd:YAG laser posterior capsulotomy if the capsular bag distension was still present.

The patients’ pupils were dilated with a 0.5% tropicamide / 0.5% phenylephrine solution 30 minutes before capsulotomy. The capsulotomy was performed with the aid of an Abraham capsulotomy lens using topical anesthesia with 0.5% proparacaine hydrochloride drops. All capsulotomies were performed by the same clinical expert (YK Han). All patients were prescribed 1% prednisolone acetate drops for 1 week following surgery. Post-capsulotomy IOP was measured by pneumatic tonometer at 1 hour after capsulotomy. 0.5% apraclonidine, which has no significant effect in the anterior chamber depth, pupil size and refraction [10,11], was prescribed when post-capsulotomy IOP was elevated more than 21mmHg. Follow-up clinical examinations were repeated at 1 week and 1 month after capsulotomy. AXL and ACD were measured 1 week after laser capsulotomy.

Data was analyzed by the Wilcoxon Signed-Rank test and Mann-Whitney test using SPSS (version 18.0, SPSS Inc., Chicago, USA). A *P* value less than 0.05 was considered statistically significant.

## Results

Table 1 shows clinical data obtained from 22 eyes from 20 patients who had been diagnosed with late postoperative CBDS and had undergone laser capsulotomy.

**Table 1:** Clinical data of patients with late postoperative capsular bag distension syndrome.

Parameter		
Male : Female		11 : 9
Mean age of onset (y) ± SD		68.6 ± 9.8
Onset time after cataract surgery (y) ± SD		6.5 ± 2.2
Right : Left : Bilateral		9 : 9 : 2
Mean UCVA (logMAR) ± SD		0.51 ± 0.28
Mean IOP (mmHg) ± SD		13.1 ± 3.2
Mean AXL(mm) ± SD	By IOLMaster	23.78 ± 2.17
Mean ACD(mm) ± SD	By Ultrabiomicroscopy	4.21 ± 0.39
Ophthalmic viscosurgical devices	Cohesive	15 (17 eyes)
	Dispersive	4
	Unknown	1
Intraocular lens type	Hydrophobic acryl, 3 pieces	16 (18 eyes)
	Hydrophobic acryl, 1 piece	2
	PMMA, 3 pieces	1
	Unknown	1

ACD = anterior chamber depth; AXL = axial length; IOP = intraocular pressure; LogMAR = logarithm of the minimum angle of resolution; PMMA = polymethylmethacrylate; SD = standard deviation; UCVA = uncorrected visual acuity

In the majority of the patients, Sensar AR40e (51.9%; Abbott Medical Optics, Inc., California, USA) and Healon (77.2%; Abbott Medical Optics, Inc., California, USA) were used for the cataract surgery.

The mean UCVA was 0.49 ± 0.24 logMAR (range, 0.02 to 1.0) before capsulotomy and 0.76 ± 0.18 logMAR (range, 0.5 to 1.0) after capsulotomy. Post-capsulotomy IOP was increased to more than 30 mmHg in 3 eyes; the use of single topical glaucoma medication resulted in IOP normalization within 1 week of the capsulotomy in these patients. Other complications such as severe anterior chamber inflammation, cystoid macular edema or retinal detachment were not evident.

Anterior capsulotomy was performed on 5 eyes from 5 patients. For 3 patients, late postoperative CBDS was successfully resolved by anterior capsulotomy alone. For one patient who had a highly myopic eye with long AXL (preoperative AXL = 32.62 mm) and a deep anterior chamber (preoperative ACD = 4.14 mm), posterior capsulotomy was successfully performed on the anteriorly relocated posterior capsule after partial removal of the accumulated fluid by anterior capsulotomy. This two-stage technique resulted in a UCVA improvement of 0.6 logMAR with a considerable degree of hyperopic shift (spherical equivalent change: +5.8 diopter [D]) without complications. Posterior capsulotomy was required in another patient because of recurrent CBDS after anterior laser capsulotomy. Initially, late postoperative CBDS was successfully treated by anterior capsulotomy, with UCVA improving by 0.4 logMAR with a hyperopic shift from -3.88 to -1.75 D. However CBDS recurrence was observed 10 months later. After posterior capsulotomy, the UCVA improved by 0.2 logMAR with a concurrent decrease in hyperopic shift from -5.00 to -1.50 D.

Posterior capsulotomy was used as the primary treatment for 17 eyes from 16 patients. Two patients complained of floaters after posterior capsulotomy, however the symptom persisted for less than 1 week. The results of anterior and posterior capsulotomy are compared in Table 2.

**Table 2:** Results of anterior or posterior capsulotomy for the late postoperative capsular bag distension syndrome.

	Anterior Capsulotomy	Posterior Capsulotomy
Number of eyes	3	17
UCVA change (logMAR) ± SD	+0.40 ± 0.36	+0.24 ± 0.16
Refractive error change (D) ± SD	+0.88 ± 0.66	+0.85 ± 0.85
IOP >30 mmHg	1 (33.0%)	2 (11.8%)
IOP normalization at 1 week	100%	100%

D = diopter; IOP = Intraocular pressure; LogMAR = logarithm of the minimum angle of resolution; UCVA = uncorrected visual acuity

**Fig 1:**
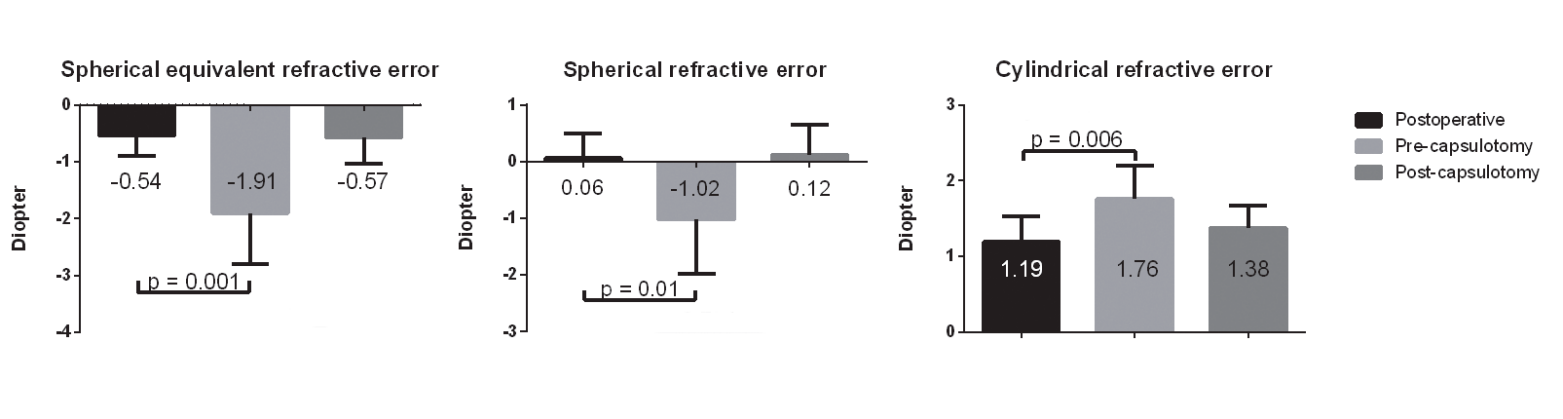
Refractive status in late postoperative capsular bag distension syndrome. The averaged refractive error in late postoperative capsular bag distension syndrome were shown with 95% confidence interval. Each measurement was performed at 2 months after cataract surgery (postoperative), before laser capsulotomy (pre-capsulotomy), and 1 week after laser capsulotomy (post-capsulotomy).

Most of the patients exhibited a late postoperative CBDS induced myopic shift: 16 of 20 eyes showed myopic changes (spherical refractive error [Dsph] change = -1.96 D; range, -6.00 to -0.25 D) and 4 of 20 eyes showed hyperopic changes (Dsph change = +0.66 D; range, +0.25 to +1.00 D). The average spherical equivalent refractive error changes associated with the development of late postoperative CBDS were -1.37 D in our study. Cylindrical refractive errors also increased as a result of late postoperative CBDS development; these were restored to postoperative values by laser capsulotomy. The averaged refractive status and 95% confidence intervals (CI) at 2 months post cataract surgery, before capsulotomy, and 1 week after capsulotomy are compared in Fig 1.

**Fig 2:**
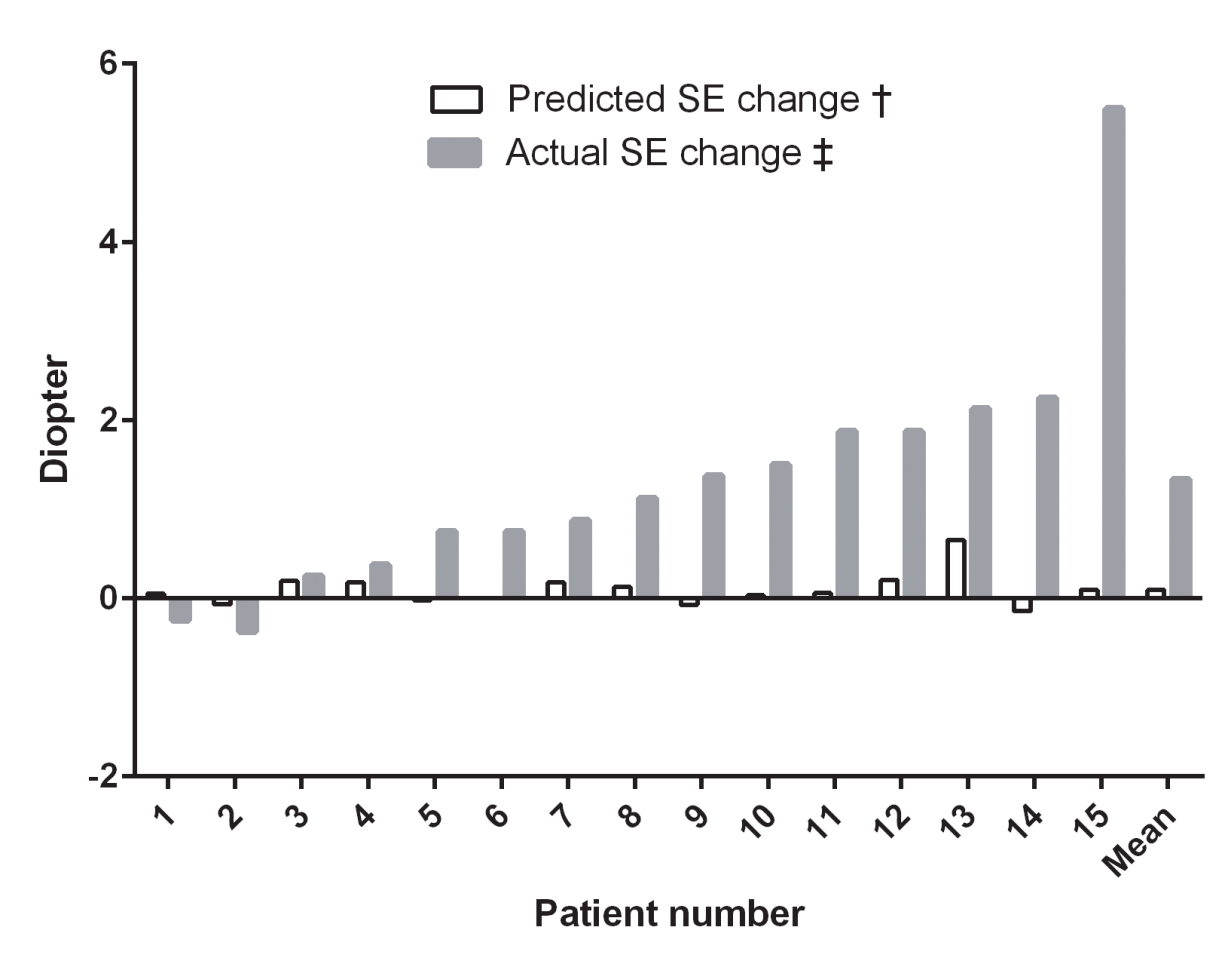
Comparison of spherical equivalent changes in late postoperative capsular bag distension syndrome. Predicted spherical equivalent change and the actual refractive error change induced by laser capsulotomy in late postoperative capsular bag distension syndrome were compared in each patient. Difference between refractive error changes suggests that refractive material in capsular bag may play a major role in the myopic shift.

Before laser capsulotomy, UBM evaluation revealed an average ACD of 4.18 mm (range, 3.40 to 5.03 mm). After laser capsulotomy the average ACD change was +0.04 mm (95% CI, +0.01 to +0.06 mm, p = 0.034) with an average depth of 4.22 mm. The averaged IOL displacement equates to +0.10 D of predicted refractive error change [5], quite different from actual spherical equivalent refraction difference according to capsulotomy (+1.33 D). Fig 2 compares the predicted refractive error change based on UBM data and the actual refractive change in 15 eyes.

To assess the relationship between biometric properties and the development of late postoperative CBDS, we compared preoperative AXL and ACD in eyes with CBDS with their fellow eyes in bilaterally pseudophakic patients. These phakic properties were measured by IOL master with sufficient repeatability [12]. Operator, surgical techniques, surgical instruments, types of IOL, and OVD were used as control variables for this comparison. Most patients had undergone operations in both eyes within a 1-week period, except 1 patient who underwent surgery in the affected eye 5 years earlier than the fellow eye. The results of this comparison are shown in Table 3.

**Table 3:** Axial length and anterior chamber depth measured by IOLMaster for both pseudophakic eyes with unilateral capsular bag distension syndrome.

	Eye with CBDS	Fellow eye	Difference	p value
Preoperative ACD (n = 4)	3.68 mm	3.44 mm	+0.24 mm	0.068
Preoperative AXL (n = 10)	23.45 mm	23.43 mm	+0.02 mm	0.449

ACD = anterior chamber depth; AXL = axial length; CBDS = capsular bag distension syndrome.

Preoperative ACD was deeper in the eye with CBDS in all patients; however, the difference in preoperative AXL between the two eyes was not significantly different (mean AXL difference—0.01 mm).

## Discussion

Nd:YAG laser capsulotomy was a successful treatment that achieved resolution of capsular bag distension resulting in visual acuity improvement in the majority of our patients. The complications of laser capsulotomy observed in this study were vitreous floaters and increased IOP; the former resolved spontaneously, while the latter resolved after administration of topical medication.

In some cases, anterior laser capsulotomy is chosen as the primary treatment option for late postoperative CBDS. The authors of several studies have cautioned about potential complications after posterior capsulotomy, such as cystoid macular edema and retinal detachment [5,13,14]. Anterior capsulotomy, a preferred technique when targeting the posterior capsule, was difficult because of media opacity or severe vaulting of the posterior capsule.

A low overall success rate, capsular wrinkling, and the risk of IOP elevation can be disadvantages of anterior capsulotomy. According to Durak et al., the success rate after anterior and posterior capsulotomy was 50% (5 of 10) and 100% (5 of 5) in early CBDS cases [14]. Marked wrinkling of the posterior capsule after anterior capsulotomy, which may be a result of redundancy due to long-standing capsular distension, was reported in late postoperative CBDS cases [15,16]. Increased IOP and CBDS recurrence after anterior capsulotomy has been reported by Colakoglu et al. [17]. In this late postoperative CBDS case, posterior capsulotomy was hindered by a pool of homogeneous milky fluid and white fragments in the hyperdistended capsular bag. Following anterior capsulotomy, the elevation of the IOP was not controlled by medical treatment; therefore, vigorous bimanual irrigation and aspiration was performed 2 days after the capsulotomy. One-month later, capsular distension recurred, and posterior capsulotomy was immediately performed. The authors suggested that posterior capsulotomy is a safer procedure for the treatment of late postoperative CBDS.

In our patients, the proportion with increased IOP after capsulotomy was greater in the anterior capsulotomy group (1 of 3) than in the posterior capsulotomy group (2 of 17). CBDS recurrence at 10 months after anterior capsulotomy was reported in 1 case. The distension had only been partially resolved by the initial anterior capsulotomy monotherapy in another case; as a consequence additional posterior capsulotomy was required immediately after anterior capsulotomy. This 2-stage technique successfully accomplished the goal of the treatment.

On the basis of our results, we recommend posterior laser capsulotomy as a primary treatment for late postoperative CBDS, even when PCO is present. If posterior capsulotomy fails because of opaque fluid in the pocket or severe vaulting of the posterior capsule, a 2-stage technique or surgical drainage can be effective alternative treatment options.

In contrast to early postoperative CBDS, previous studies have suggested that late postoperative CBDS may be less likely to be associated with induced myopia. Omar et al. reported a hyperopic shift of +3.60 D after capsulotomy for early postoperative CBDS [4]. This finding was explained by anterior displacement of the IOL due to capsular bag extension. Sorenson et al. showed that mean predicted refractive status based on IOL movement (0.75 mm deeper after capsulotomy) was not different from actual myopic changes during early CBDS development (-1.23 vs. -1.50 D; p = 0.29) [5]. However, Landa et al. reported a refractive error change of <0.25 D in 87.5% of late postoperative CBDS patients following laser capsulotomy [6]. According to Pinarci et al., capsulotomy did not change the refractive error in 14 of 15 eyes (93.3%) affected by late postoperative CBDS [7].

Laser capsulotomy successfully reversed refractive error changes, which resulted in similar post-capsulotomy and postoperative refractive errors (Fig 1). Two possible explanations have been previously suggested for the refractive status changes in CBDS, IOL displacement and the lens effects of opalescent material in the distended capsular bag. We assessed the former using UBM and found only a minimal increase in ACD after laser capsulotomy. This finding suggests that opalescent material in the capsular bag, which is less evident in early postoperative CBDS, may play a major role in the myopic shift of late postoperative CBDS [5].

Rigid fibrotic changes in capsular structure may result in posterior capsular distension rather than anterior IOL displacement [7,16,18]. The increased refractive index of the chronically condensed opaque capsular bag fluid is able to generate convergence power when the curvature of the distended posterior capsule exceeds that of the posterior IOL surface. Concave lens effects that result in hyperopic shift could also arise as a result of an inverse relationship between the steepness of the posterior capsule and the posterior IOL surface. Similarly, tangentially unequal distension of the posterior capsule could be an explanation for CBDS induced astigmatism.

Preoperative AXL of the affected eye in late postoperative CBDS shows no significant difference to the AXL in the fellow eye. The p value for the preoperative ACD comparison was smaller than that for the AXL comparison, although it did not reach statistical significance due to the small sample size. These findings suggest that a deep ACD rather than a long AXL influences the development of late postoperative CBDS. Late postoperative CBDS is thought to occur because of metaplasia and proliferation of lens epithelial cells, which leads to the accumulation of various types of collagen and extracellular matrix material in the capsular bag, leading to the osmotic influx of fluid [1,18,19]. Removal of the OVD and epithelial cells in the capsular bag can be a lengthy process when the anterior chamber is deep, and removal may be incomplete as a result.

The follow-up period after uneventful cataract surgery was usually less than 1 year in our hospital setting. Because pseudophakic patients have a similar follow-up period to late postoperative CBDS patients, we considered using them as a control group. However, we were unable to recruit sufficient numbers of pseudophakic patients to act as a suitable control group and hence were forced to use the fellow eye of the patient as the control. Owing to the differences in group size and criteria, our results cannot be used to refute the finding of a previous study on early postoperative CBDS, which suggests that a long AXL (>25 mm) is a risk factor for the condition [19].

In conclusion, late postoperative CBDS results from changes in the refractive status due to the lens effect of opaque material in the distended capsular bag rather than anterior displacement of IOL. This refractive error change can be successfully treated by Nd:YAG laser posterior capsulotomy without serious complications. The relationship of anterior segmental structure parameters, such as ACD and the development of late postoperative CBDS, merits further investigation.
